# Genome Sequence of Lactobacillus plantarum KB1253, a Gamma-Aminobutyric Acid (GABA) Producer Used in GABA-Enriched Tomato Juice Production

**DOI:** 10.1128/MRA.00158-19

**Published:** 2019-07-18

**Authors:** Yuki Nakatani, Masanori Fukao, Tetsuya Fukaya

**Affiliations:** a Innovation Division, Kagome Co., Ltd., Nasushiobara, Tochigi, Japan; University of Delaware

## Abstract

Here, we present the draft genome sequence of Lactobacillus plantarum KB1253, isolated from a traditional Japanese pickle. Its genome comprises 3,097 genes and 3,305,456 nucleotides, with an average G+C content of 44.4%.

## ANNOUNCEMENT

Gamma-aminobutyric acid (GABA), a 4-carbon nonproteinaceous amino acid found ubiquitously in nature, is associated with several well-characterized physiological functions (1–4). Many microorganisms can produce GABA, including bacteria, fungi, and yeasts, although lactic acid bacteria (LAB) are the most utilized GABA producers (5–8). GABA-producing LAB have been a focus of research in recent years because they possess unique physiological properties and are regarded as safe for use in the food industry (6, 9–11). Many food-derived lactobacilli produce GABA, including several strains of Lactobacillus brevis (12–16), L. plantarum (17–21), L. buchneri (6, 22–24), L. paracasei (13, 18, 21, 25), and single strains of L. rhamnosus (21, 25), L. delbrueckii (18, 25), and other species (25–29). Currently, there are no reports on the genetic basis of the technological and safety aspects of lactobacilli used in GABA-enriched tomato juice production.

We diluted a traditional Japanese pickle in saline, incubated the diluted solution on a de Man-Rogosa-Sharpe (MRS) agar (Oxoid, Cambridge, UK) plate for 24 h at 30°C, and obtained a single colony of strain KB1253. Genomic DNA was isolated from this single colony, which was grown in MRS broth overnight at 30°C under anaerobic conditions. DNA was extracted using a Genomic-tip 500/G kit (Qiagen, Valencia, CA). Detailed genome analyses will help elucidate the genetic basis of GABA production, favorable flavor, and food safety.

Libraries for sequencing were prepared at Genewiz, Inc. (South Plainfield, NJ), using a Nextera XT DNA library preparation kit (Illumina, San Diego, CA) following the manufacturer’s recommended procedures. The sequencing libraries were sequenced on an Illumina MiSeq instrument using the MiSeq reagent kit version 2 for 2 × 250-bp paired-end reads. Sequence reads were trimmed to remove nucleotides with poor quality at the 3′ end (error rate, <0.05). If there were two nucleotides with poor quality (error rate, <0.05), these nucleotides and all downstream nucleotides were removed. After trimming, reads with <100 bases were removed from subsequent assembly. A total of 30,678,684 reads were generated and assembled into 82 contigs using CLC Genomics Workbench 9.0 (Qiagen, Valencia, CA), with an *N*_50_ value of 120,850 bp and average read coverage of 1,654-fold. The 16S rRNA sequence from the assembled genome was compared to the NCBI 16S rRNA database via a blastn search, identifying the best hit to the Lactobacillus plantarum subsp. *plantarum* NBRC 15891 sequence (GenBank accession number NR_113338; query coverage, 100%; identity, 99.93%). The genome annotation was performed by the DDBJ Fast Annotation and Submission Tool (DFAST) (30), an annotation server optimized for Lactobacillus spp. (6). The default settings were used. The final sequence is composed of 3,305,456 bp with 3,097 putative open reading frames (ORFs) and a G+C content of 44.4%.

The genome of strain KB1253 contains two *gadB* genes coding for glutamate decarboxylase. One is located next to *gadC* and likely regulated as a whole operon with *gts* (glutamyl-tRNA synthetase) and *gadR* (transcriptional regulator), as reported for L. brevis strains (31, 32); the other gene is located far from the operon. These genes may be involved in GABA synthesis in this strain. The genome also revealed the presence of gene clusters homologous to genes associated with the production of bacteriocins such as plantaricin.

We also surveyed the KB1253 genome for genes related to transferable antibiotic resistance and virulence factors, according to a previous report (33); however, as with previously sequenced *L. plantarum* strains, none were found. These data, supported by *in vivo* studies, confirm that KB1253 can be considered “generally recognized as safe” (GRAS) with “qualified presumption of safety” (QPS) status.

The genome sequence of *L. plantarum* KB1253 is a promising resource for further identification of genes involved in beneficial processes, such as the production of GABA and other metabolites related to favorable flavor during fermentation with tomato juice. In addition, these data contribute to the availability of GABA-producing *Lactobacillus* genome sequences and enable a better understanding of their potential technological and biofunctional properties.

### Data availability.

This whole-genome sequence was deposited at DDBJ/EMBL/GenBank under the accession numbers BIFE01000001 to BIFE01000082. The version described in this paper is the first version, BIFE01000000. Raw data were deposited in the Sequence Read Archive (SRA) under BioSample number SAMN11080842 and SRA run number SRR8693953, which are part of SRA study number SRP187802.

## References

[1] Pouliot Mathieu K, Gardner Fortier C, Lemieux S, St Gelais D, Champagne CP, Vuillemard JC 2013 Effect of cheese containing gamma-aminobutyric acid-producing lactic acid bacteria on blood pressure in men. PharmaNutrition 1:141–148. doi:10.1016/j.phanu.2013.06.003.

[2] Parkash J, Kaur G 2007 Potential of PSA-NCAM in neuron-glial plasticity in the adult hypothalamus: role of noradrenergic and GABAergic neurotransmitters. Brain Res Bull 74:317–328. doi:10.1016/j.brainresbull.2007.07.005. 17845906

[3] Han D, Kim H-Y, Lee H-J, Shim I, Hahm D-H 2007 Wound healing activity of gamma-aminobutyric acid (GABA) in rats. J Microbiol Biotechnol 17:1661–1669. 18156782

[4] Wong CG, Bottiglieri T, Snead OC 2003 GABA, gamma-hydroxybutyric acid, and neurological disease. Ann Neurol 54:S3–S12. doi:10.1002/ana.10696. 12891648

[5] Plokhov AY, Gusyatiner MM, Yampolskaya TA, Kaluzhsky VE, Sukhareva BS, Schulga AA 2000 Preparation of γ-aminobutyric acid using *E. coli* cells with high activity of glutamate decarboxylase. Appl Biochem Biotechnol 88:257–265. doi:10.1385/ABAB:88:1-3:257.

[6] Komatsuzaki N, Shima J, Kawamoto S, Momose H, Kimura T 2005 Production of γ-aminobutyric acid (GABA) by *Lactobacillus paracasei* isolated from traditional fermented foods. Food Microbiol 22:497–504. doi:10.1016/j.fm.2005.01.002.

[7] Jiang D, Cai Q, Gao A, Li J, Yang Y, Xu X, Ye Y, Hou J 2013 Cloning and expression of a full-length glutamate decarboxylase gene from a high-yielding γ-aminobutyric acid yeast strain MJ2. Ann Microbiol 63:487–494. doi:10.1007/s13213-012-0493-9.

[8] Cai S, Gao F, Zhang X, Wang O, Wu W, Zhu S, Zhang D, Zhou F, Ji B 2014 Evaluation of γ-aminobutyric acid, phytate and antioxidant activity of tempeh-like fermented oats (*Avena sativa* L.) prepared with different filamentous fungi. J Food Sci Technol 51:2544–2551. doi:10.1007/s13197-012-0748-2. 25328194 PMC4190224

[9] Kook M-C, Cho S-C, Kang J, Song Y, Park H 2014 Effect of gamma-aminobutyric acid produced by *Lactobacillus sakei* B2-16 on diet and exercise in high fat diet-induced *Obese* rats. Food Sci Biotechnol 23:1965–1970. doi:10.1007/s10068-014-0268-0.

[10] Park S-Y, Lee J-W, Lim S-D 2014 The probiotic characteristics and GABA production of *Lactobacillus plantarum* K154 isolated from kimchi. Food Sci Biotechnol 23:1951–1957. doi:10.1007/s10068-014-0266-2.

[11] Lee J-Y, Jeon S-J 2014 Characterization and immobilization on nickel-chelated Sepharose of a glutamate decarboxylase A from *Lactobacillus brevis* BH2 and its application for production of GABA. Biosci Biotechnol Biochem 78:1656–1661. doi:10.1080/09168451.2014.936347. 25047135

[12] Villegas JM, Brown L, Savoy de Giori G, Hebert EM 2016 Optimization of batch culture conditions for GABA production by *Lactobacillus brevis* CRL 1942, isolated from quinoa sourdough. LWT 67:22–26. doi:10.1016/j.lwt.2015.11.027.

[13] Wu Q, Shah NP 2015 Gas release-based prescreening combined with reversed-phase HPLC quantitation for efficient selection of high-γ-aminobutyric acid (GABA)-producing lactic acid bacteria. J Dairy Sci 98:790–797. doi:10.3168/jds.2014-8808. 25497828

[14] Binh TTT, Ju W-T, Jung W-J, Park R-D 2014 Optimization of γ-amino butyric acid production in a newly isolated *Lactobacillus brevis*. Biotechnol Lett 36:93–98. doi:10.1007/s10529-013-1326-z. 24078124

[15] Li H, Cao Y 2010 Lactic acid bacterial cell factories for gamma-aminobutyric acid. Amino Acids 39:1107–1116. doi:10.1007/s00726-010-0582-7. 20364279

[16] Dhakal R, Bajpai VK, Baek K-H 2012 Production of GABA (γ-aminobutyric acid) by microorganisms: a review. Braz J Microbiol 43:1230–1241. doi:10.1590/S1517-83822012000400001. 24031948 PMC3769009

[17] Di Cagno R, Mazzacane F, Rizzello CG, De Angelis M, Giuliani G, Meloni M, De Servi B, Gobbetti M 2010 Synthesis of gamma-aminobutyric acid (GABA) by *Lactobacillus plantarum* DSM19463: functional grape must beverage and dermatological applications. Appl Microbiol Biotechnol 86:731–741. doi:10.1007/s00253-009-2370-4. 20013120

[18] Siragusa S, De Angelis M, Di Cagno R, Rizzello CG, Coda R, Gobbetti M 2007 Synthesis of γ-aminobutyric acid by lactic acid bacteria isolated from a variety of Italian cheeses. Appl Environ Microbiol 73:7283–7290. doi:10.1128/AEM.01064-07. 17890341 PMC2168214

[19] Park K-B, Oh S-H 2004 Cloning and expression of a full-length glutamate decarboxylase gene from *Lactobacillus plantarum*. J Food Sci Nutr 9:324–329. doi:10.3746/jfn.2004.9.4.324.

[20] Tajabadi N, Baradaran A, Ebrahimpour A, Rahim RA, Bakar FA, Manap MYA, Mohammed AS, Saari N 2015 Overexpression and optimization of glutamate decarboxylase in *Lactobacillus plantarum* Taj-Apis362 for high gamma-aminobutyric acid production. Microb Biotechnol 8:623–632. doi:10.1111/1751-7915.12254. 25757029 PMC4476817

[21] Franciosi E, Carafa I, Nardin T, Schiavon S, Poznanski E, Cavazza A, Larcher R, Tuohy KM 2015 Biodiversity and *γ* -aminobutyric acid production by lactic acid bacteria isolated from traditional alpine raw cow’s milk cheeses. Biomed Res Int 2015:1–11. doi:10.1155/2015/625740. PMC4352725 25802859

[22] Zhao A, Hu X, Pan L, Wang X 2015 Isolation and characterization of a gamma-aminobutyric acid producing strain *Lactobacillus buchneri* WPZ001 that could efficiently utilize xylose and corncob hydrolysate. Appl Microbiol Biotechnol 99:3191–3200. doi:10.1007/s00253-014-6294-2. 25524701

[23] Cho YR, Chang JY, Chang HC 2007 Production of gamma-aminobutyric acid (GABA) by *Lactobacillus buchneri* isolated from kimchi and its neuroprotective effect on neuronal cells. J Microbiol Biotechnol 17:104–109. 18051360

[24] Park KB, Oh SH 2006 Isolation and characterization of *Lactobacillus buchneri* strains with high gamma-aminobutyric acid producing capacity from naturally aged cheese. Food Sci Biotechnol 15:86–90.

[25] Seok J-H, Park K-B, Kim Y-H, Bae M-O, Lee M-K, Oh S-H 2008 Production and characterization of kimchi with enhanced levels of γ-aminobutyric acid. Food Sci Biotechnol 17:940–946.

[26] Park JY, Jeong S-J, Kim JH 2014 Characterization of a glutamate decarboxylase (GAD) gene from *Lactobacillus zymae*. Biotechnol Lett 36:1791–1799. doi:10.1007/s10529-014-1539-9. 24770872

[27] Wang Y, Wang Y, Lang C, Wei D, Xu P, Xie J 2015 Genome sequence of *Lactobacillus curieae* CCTCC M 2011381^T^, a novel producer of gamma-aminobutyric acid. Genome Announc 3:e00552-15. doi:10.1128/genomeA.00552-15. 26021929 PMC4447914

[28] Sanchart C, Rattanaporn O, Haltrich D, Phukpattaranont P, Maneerat S 2016 Technological and safety properties of newly isolated GABA-producing *Lactobacillus futsaii* strains. J Appl Microbiol 121:734–745. doi:10.1111/jam.13168. 27147524

[29] Sa HD, Park JY, Jeong S-J, Lee KW, Kim JH 2015 Characterization of glutamate decarboxylase (GAD) from *Lactobacillus sakei* A156 isolated from jeot-gal. J Microbiol Biotechnol 25:696–703. doi:10.4014/jmb.1412.12075. 25791853

[30] Tanizawa Y, Fujisawa T, Kaminuma E, Nakamura Y, Arita M 2016 DFAST and DAGA: Web-based integrated genome annotation tools and resources. Biosci Microbiota Food Health 35:173–184. doi:10.12938/bmfh.16-003. 27867804 PMC5107635

[31] Yunes RA, Poluektova EU, Dyachkova MS, Klimina KM, Kovtun AS, Averina OV, Orlova VS, Danilenko VN 2016 GABA production and structure of *gadB*/*gadC* genes in *Lactobacillus* and *Bifidobacterium* strains from human microbiota. Anaerobe 42:197–204. doi:10.1016/j.anaerobe.2016.10.011. 27794467

[32] Li H, Li W, Liu X, Cao Y 2013 *gadA* gene locus in *Lactobacillus brevis* NCL912 and its expression during fed-batch fermentation. FEMS Microbiol Lett 349:108–116. doi:10.1111/1574-6968.12301. 24164637

[33] Fukao M, Oshima K, Morita H, Toh H, Suda W, Kim S-W, Suzuki S, Yakabe T, Hattori M, Yajima N 2013 Genomic analysis by deep sequencing of the probiotic *Lactobacillus brevis* KB290 harboring nine plasmids reveals genomic stability. PLoS One 8:e60521. doi:10.1371/journal.pone.0060521. 23544154 PMC3609814

